# Viruses Beyond Pathogens: Partners and Tools for Biotechnology

**DOI:** 10.3390/v17101401

**Published:** 2025-10-21

**Authors:** Patrick Materatski, Carla Varanda

**Affiliations:** 1 MED—Mediterranean Institute for Agriculture, Environment and Development & CHANGE—Global Change and Sustainability Institute, University of Évora, Largo dos Colegiais 2, 7004-516 Évora, Portugal; 2 Research Centre for Natural Resources, Environment and Society (CERNAS), Santarém Polytechnic University, School of Agriculture, Quinta do Galinheiro-S. Pedro, 2001-904 Santarém, Portugal

## 1. Introduction

For many years, viruses were regarded solely as agents of devastating diseases in humans, animals, and plants. While it is true that viruses have caused immense suffering and death throughout history, they have also shown us that they sustain life in profound ways. Fragments of ancient viral genomes, known as endogenous retroviruses, were integrated into the microbiome of our ancestors millions of years ago and now comprise approximately 8% of our DNA. These viral sequences influence reproduction and neurodevelopment, regulating essential processes such as embryonic development, immunity, and cell differentiation [1].

Viruses have also proven to be allies in several other aspects crucial to human survival. In 1917, while studying dysentery caused by *Shigella dysenteriae*, the microbiologist Félix d’Hérelle noticed a striking difference between samples from the patients who recovered and those who did not; in the survivors, invisible agents, which he named bacteriophages, destroyed the bacteria [2]. In 1919, recognizing the therapeutic potential of bacteriophages, d’Hérelle isolated phages targeting Salmonella during a typhoid outbreak in poultry and successfully cured the infected birds [3].

It was not until the invention of the electron microscope in 1940 that the true nature of bacteriophages was revealed: viruses that infect bacteria and archaea, leaving plants and animals unharmed [4]. Many studies, such as d’Hérelle’s work, as well as other pioneering efforts such as the use of cowpox to protect against smallpox in the 18th century and the use of viruses to develop vaccines against many other important diseases [5], laid the groundwork for recognizing viruses not only as pathogens, but also as powerful tools for medicine and scientific discovery. This led to the rapid development of mRNA vaccines during the COVID-19 pandemic [6], and the trajectory of virus-based technologies mirrors the progress of science itself. Viruses are increasingly being redefined as versatile tools for biotechnology, and their ubiquity, structural sophistication, small genomes, and genetic adaptability have positioned them not only as agents of disease but also as engines of innovation [7]. In recent decades, advances in genomics, synthetic biology, and molecular engineering have significantly enhanced our ability to detect, sequence, and modify viruses with unprecedented speed and precision [8,9]. Today, viruses serve as highly efficient delivery systems in gene therapy [10], precision tools in genome editing [11], biocontrol agents in agriculture [12], and nanoscale building blocks in materials science [13]. For instance, despite their use decreasing due to the golden era of antibiotics, bacteriophages are now regaining interest as targeted antimicrobial agents in alternative therapies against antibiotic-resistant bacteria [14].

A new era of viral biotechnology is emerging at the intersection with artificial intelligence (AI). AI-powered algorithms are accelerating viral genome annotation, predicting host–virus interactions, and guiding the rational design of viral vectors with improved safety and efficacy [15,16]. Machine learning models can simulate viral evolution, identify optimal targets for intervention, and optimize the structural design of virus-like particles for vaccines and nanomaterials [17,18]. This synergy between AI and virology holds the promise of reducing development timelines from years to months, enabling rapid responses to emerging pathogens and expanding the repertoire of beneficial viral applications [19].

While it remains essential to acknowledge the considerable harm viruses can cause, it is equally important to recognize the extraordinary opportunities they present when approached with the right tools and vision. This Special Issue, “The Application of Viruses to Biotechnology 3.0”, brings together cutting-edge research that reflects this paradigm shift, showing how viruses, once seen almost exclusively as threats, are now pivotal to some of the most exciting advances in modern science.

## 2. Special Issue Overview

Building on the success of our previous Special Issues, “The Application of Viruses to Biotechnology” (2021), which collated 16 articles and has since accumulated over 385 citations, and “Insights into the Application of Viruses to Biotechnology” (2022), comprising 9 contributions, we are pleased to introduce the third segment in this series: “Advances in the Application of Viruses to Biotechnology”.

This edition collates six contributions—two research articles, one communication, two reviews, and one brief report—that highlight the most up-to-date research on the use of viruses in biotechnology. The contributions are listed below:

1. Banos-Mateos, S., Lopez-Robles, C., Yubero, M. E., Jurado, A., Arbelaiz-Sarasola, A., Lamsfus-Calle, A., Arrasate, A., Albo, C., Ramírez, J. C., & Fertin, M. J. (2024). Abolishing Retro-Transduction of Producer Cells in Lentiviral Vector Manufacturing. Viruses, 16(8), 1216. https://doi.org/10.3390/v16081216.
2. Falcón, A., Martínez-Pulgarín, S., López-Serrano, S., Reytor, E., Cid, M., Nuñez, M. d. C., Córdoba, L., Darji, A., & Escribano, J. M. (2024). Development of a Fully Protective Pandemic Avian Influenza Subunit Vaccine in Insect Pupae. Viruses, 16(6), 829. https://doi.org/10.3390/v16060829.
3. Slack, J., Nguyen, C., & Ibe-Enwo, A. (2024). A Lac Repressor-Inducible Baculovirus Expression Vector for Controlling Adeno-Associated Virus Capsid Ratios. Viruses, 16(1), 51. https://doi.org/10.3390/v16010051.
4. Bakhshinejad, B., & Ghiasvand, S. (2025). A Beautiful Bind: Phage Display and the Search for Cell-Selective Peptides. Viruses, 17(7), 975. https://doi.org/10.3390/v17070975.
5. Schieferecke, A. J., Kuxhausen Ralph, N., & Schaffer, D. V. (2025). The Application of DNA Viruses to Biotechnology. Viruses, 17(3), 414. https://doi.org/10.3390/v17030414.
6. López-Vidal, J., Martínez-Pulgarín, S., Martínez-Alonso, D., Cid, M., & Escribano, J. M. (2024). Enhanced Recombinant Protein Expression in Insect Cells by Natural and Recombinant Components of Lepidoptera Hemolymph. Viruses, 16(6), 944. https://doi.org/10.3390/v16060944.

Viral vectors are mainly based on adenoviruses, adeno-associated viruses (AAVs), and retroviruses, each with unique characteristics for specific gene therapy applications [5]. Adenoviruses present the largest DNA loading capacity but also high immunogenicity in the human population. AAVs are the most commonly used viral vectors and are well standardized; however, they have a low DNA loading capacity, may cause off-target infections, and require repetitive administrations to maintain therapeutic effect, potentially resulting in patient immunity. Retroviruses have a potent therapeutic effect due to the integration of the transgene into the host genome, but may raise concerns in terms of safety due to potential oncogene activation. Among them, lentiviral vectors (LVVs), derived from HIV-1, represent a type of retrovirus that can load large genetic material. They have been engineered to increase their safety and present a low risk of oncogene activation, despite their integrative nature [20]. Manufacturing demand for LVVs has increased in recent years due to their uses in many cell therapies and clinical trials [21]. However, LVV manufacture faces important challenges, such as retrotransduction of producer cells, a factor responsible for reduced yield in lentiviral vector production (loss of 70–90% of viable particles). In this sense, the study by Banos-Mateos (Contribution 1) explores this phenomenon by quantifying its extent and testing different mitigating strategies focusing on the interaction between the VSV-G envelope protein and the LDLR receptor. The authors show that the prevention of viral re-entry is possible. The work reveals that, despite their promise, approaches to blocking retrotransduction can have side effects on cellular metabolism, affecting overall productivity. It is therefore essential to weigh the gains and losses to ensure that the increase in LVV production compensates for the metabolic hurdles caused by these approaches.

Avian influenza viruses have caused thousands of human deaths and continuous disease outbreaks in domestic poultry and wild birds [22]. Vaccines are the most effective measure against influenza, but it is necessary they become available in time to diminish the possibility of a pandemic and that they are not hindered by limitations in global production capacity. Influenza vaccines can be developed using distinct technologies; the present goal is to reduce dependence on egg-based approaches and embrace newer, quicker, and more scalable vaccine technologies to respond to novel influenza outbreaks and pandemics. Among these technologies, recombinant-based methods are the fastest. The work by Falcón et al. (Contribution 2) demonstrates the potential of the technology CrisBio^®^ (Tres Cantos, Spain), a technology that uses *Trichoplusia ni* pupae as natural bioreactors combined with baculoviral vectors, for the efficient production of subunit influenza vaccines. By expressing and purifying hemagglutinin (HA) of a highly pathogenic avian H7N1 virus, the authors obtained high yields and an antigen capable of fully protecting vaccinated poultry against a lethal infection. The study confirms that the produced protein maintains its immunogenicity and demonstrates cross-protection against human H7N9 viruses, offering a scalable, rapid, and effective platform for the development of pandemic vaccines, with potential for use in both animal and human health.

The study by Falcón et al. (Contribution 2) shows the use of insects in baculoviral vector production, and the study by López-Vidal (Contribution 6) shows that lepidoptera hemolymph proteins can increase baculoviral vector production in insect cell cultures. López-Vidal (Contribution 6) demonstrates that supplementing media with *Trichoplusia ni* hemolymph increases the yield of recombinant proteins (≈1.5-fold) and insect cell viability (30–40% higher) and that co-expression of Bombyx mori SP2Bm with proteins of interest triples production. This approach offers an innovative way to optimize the baculovirus expression system and to increase the efficiency of recombinant protein production on a laboratory and industrial scale, reinforcing the role of insect-derived compounds as valuable tools in viral biotechnology.

The study by Slack et al. (Contribution 3) presents an innovative technology for controlling the expression of the capsid proteins VP1, VP2, and VP3 in the production of rAAV gene therapy vectors using the baculovirus expression vector (BEV). Alternative translational initiation codons for VP1 and VP2 reduce their abundances relative to VP3. The authors developed a system inducible by the Lac repressor (LacR) to regulate expression of VP1 and VP2 proteins relative to VP3 in the context of the BEV, and demonstrated that a VP1:VP2:VP3 ratio of 1:1:8 provided the optimal potency of a neurospecific rAAV9 serotype derivative. Their work showed that overexpressed VP1 does not incorporate into particles and that an excess of VP2 reduces the final rAAV titer. The study contributes to advances in viral biotechnology by offering a more controlled method for optimizing the quality and efficacy of large-scale rAAV vectors, accelerating the development of safer and more potent gene therapies.

Another example of the application of viruses to biotechnology is phage display technology. Phage display has transformed the discovery of peptides that specifically target a broad range of cell surface biomolecules, enabling modulation of numerous disease-associated protein–protein interactions at the cell membrane. Combining this technique with combinatorial chemistry has enabled the creation of phage display peptide libraries for identifying ligands to cell surface proteins through affinity selection on targets in vitro, ex vivo, or in vivo. These peptides, positioned between small molecules and large biologics, increase therapeutic options for previously undruggable targets, though library limitations and selection biases can lead to suboptimal affinity enrichment. The review article by Bakhshinejad (Contribution 4) summarizes advances in the use of phage display technology to discover peptides that selectively bind to cell surface proteins, including targets previously considered “undruggable.” By discussing in vitro, ex vivo, and in vivo selection strategies, library optimization, chemical cycling of peptides, and integration with next-generation sequencing, the work offers a practical guide to overcoming technical limitations and improving affinity and specificity. The analysis also highlights how the integration of next-generation sequencing (NGS) as well as artificial intelligence (AI) is transforming phage display into an increasingly powerful platform for the development of more precise, effective, and personalized therapies, expanding the role of viruses as key tools in modern biotechnology.

The delivery of biomolecules to precisely target cells has been a challenge in biotechnology. DNA viruses have acquired the ability to transfer genetic material and regulate cellular functions, and these characteristics have made them the focus of research as versatile tools in biotechnology. The study by Schieferecke et al. (Contribution 5) contributes to this SI with a comprehensive overview of using DNA viruses as biotechnological tools, exploring their role in basic research, health, biomanufacturing, and agriculture. The increase in virology knowledge and development of modern genetic manipulation techniques have shown how these vectors can be optimized for gene delivery and for the modulation of cellular processes, and has facilitated their use for developing vaccines, therapies, and innovative agricultural solutions. The work by Schieferecke et al. (Contribution 5) also shows the importance of balancing technological advances with ethical and biosafety concerns, contributing to the advancement of viral biotechnology by outlining ways to maximize the potential of DNA vectors safely and effectively.

## 3. Concluding Remarks

Virology and biotechnology continue to advance rapidly to drive scientific and societal progress, showing how viruses can contribute both in their natural form and as biotechnological instruments. Machine learning is also changing the pace of discoveries as it helps annotate viral genomes faster, suggests possible host–virus links, and even supports the design of new vectors and vaccines.

Endogenous viral elements show how evolution has transformed ancient infections into integral parts of our biology, reminding us that viruses are not merely agents of disease, but also drivers of innovation, adaptation, and life itself. The articles gathered in this Special Issue illustrate the breadth of viral biotechnology and point to its growing role in future developments. As Guest Editors of “The Application of Viruses to Biotechnology 3.0”, we thank the authors, reviewers, and the Editorial Office for their efforts toward making this collection possible. We hope that the research collected here will inspire new research, encourage young researchers to explore virus-based technologies, and promote synergies between biotechnology and AI. Finally, we look forward to seeing viruses continue their transformation from biological challenges into essential partners for innovation.
